# Diagnostic utility of p53 and CK20 immunohistochemical expression grading urothelial malignancies

**DOI:** 10.1186/1755-7682-7-36

**Published:** 2014-07-26

**Authors:** Shazia Mumtaz, Atif Ali Hashmi, Sheema H Hasan, Muhammad Muzzammil Edhi, Mehmood Khan

**Affiliations:** 1 Department of Histopathology, Liaquat National Hospital and Medical College, Karachi, Pakistan; 2 Department of Pathology and Microbiology, Aga Khan University Hospital, Karachi, Pakistan; 3 Liaquat National Hospital and Medical College, Karachi, Pakistan; 4 Dhaka Medical College, Dhaka, Bangladesh

**Keywords:** Papillary urothelial carcinoma, p53, Cytokeratin 20

## Abstract

**Introduction:**

Current grading system in application by WHO/ISUP divides urothelial malignancies in low and high grade by morphologic criteria while strict segregation may become cumbersome in limited tissue specimens. As grading these carcinomas are of utmost prognostic significance after depth of invasion, therefore we evaluated the role of immunohistochemical expression of p53 and cytokeratin 20 as an adjuctive tool in grading urothelial carcinoma.

**Methods:**

The study was conducted in Aga khan university hospital, Histopathology section from December 2010 till June 2011 for duration of six months. It involved 95 cases of urothelial carcinomas diagnosed on trans-uretheral resection specimens of bladder growth. Immunohistochemical expression of p53 and cytokeratin 20 was performed according to standard protocols and correlated with grade and depth of invasion.

**Results:**

There were 48 cases (50.5%) of low grade and 47 cases (49.5%) of high grade urothelial carcinoma included in the study. Male to female ratio was 4.3:1. Majority of patients (80%) were seen in 45 to 90 years age group. Diffuse positive expression of cytokerain 20 was noted in 33 cases (68.8%) of high grade and 19 (40.4%) low grade tumors. Strong positive expression of p53 was seen in 35 cases (72.9%) of high grade while only 17 cases (36.2%) of low grade tumors showed strong p53 expression.

**Conclusion:**

Significant difference in expression of Cytokeratin 20 and p53 was found between low and high grade urothelial carcinoma. Therefore we suggest combined use of these markers may be helpful in assigning grade to urothelial carcinoma especially when histologic features are borderline.

## Introduction

Bladder cancer is the second most common genitourinary malignancy with urothelial carcinomas comprising 90% of all primary bladder carcinomas. In the United States, approximately 67,000 individuals develop bladder cancer each year and 13,750 die from the disease [1]. In Pakistan, a study was carried out at the Aga Khan Hospital in which 495 cases of transitional cell carcinoma were reported during a span of three years [2]. Various risk factors are associated with development of bladder carcinomas including cigarette smoking, arylamines, aniline dyes, auramines, phenacetin, and cyclophosphomide. Schistosoma haematobium infestation, radiation exposure especially for the treatment of prostate cancer also plays a role in some cases [3].

Grading of bladder tumors is an important prognostic factor [4,5]. The first ever grading system to classify bladder tumors was proposed by Borders in 1922 [6]. The first widely used grading system was proposed by Ash in 1940 which divided bladder tumors into four grades [7]. In 1998, World Health Organization, International Society of Urological Pathology and Canadian Academy of Pathology purposed classification of urothelial tumors called WHO/ISUP Consenses Classification and classified urothelial papillary tumors into papilloma, papillary urothelial neoplasm of low malignant potential (PUNLMP), low grade carcinoma (LGC) and high grade carcinoma (HGC) [8]. Low grade carcinomas are associated with good prognosis as compared to high grade carcinoma. As further management and prognosis of patients is based on accurate grading of these tumors, therefore, this categorization is of utmost significance [9].

Multiple immunohistochemical markers particularly p53 and cytokeratin 20, have been investigated in several international studies for use as diagnostic and prognostic aids in urothelial tumors [10-12]. Being a cell proliferation regulating and pro-apoptotic gene, mutation in p53 can nullify its normal functions and increased expression of the mutant protein is regarded as a predictor of poor prognosis of urothelial tumors [4,10]. Cytokeratin 20 belongs to cytoskeleton associated with intermediate filaments, cytokeratin 20 is specifically expressed in superficial and in some intermediate cells of normal urothelium but its expression beyond these limits may suggest progression to urothelial carcinoma [13,14].

Separation of low and high grade tumors can sometimes be very difficult especially in small biopsies which may show crushing and cautry artifacts. Therefore we aimed to determine the usefulness of p53 and CK20 immunohistochemical stains as an adjunctive tool in grading urothelial carcinomas.

## Methods

It was a prospective cross-sectional study conducted in the department of histopathology, Aga khan university hospital from December 2010 till June 2011 for duration of 6 months. Patients who underwent transuretheral resection for bladder growth, subsequently diagnosed as urothelial carcinoma were included in the study. An approval from institutional ethical review committee was taken prior to conducting the study. After resection, specimens were sent to histopathology laboratory. Specimens were processed according to standard protocols and examined with routine hematoxylin and eosin sections. Urothelial carcinomas were graded according to WHO/ISUP classification of urothelial neoplasms. Cases with non-urothelial malignancies and metastatic carcinomas were excluded from the study. All cases were reviewed and graded by 2 pathologists with more than 5 years experience of reporting bladder biopsies.

### Methodology of immunohistochemistry

Immunohistochemical analysis was performed using p53 and cytokeratin 20 antibodies (DAKO, Denmark). Each assay included positive and negative controls. Paraffin sections of 3–4 micrometer thickness were placed on glass slides coated with Poly-L-Lysine (Sigma chemical Co, USA) to promote adhesion. Slides were then kept overnight at 37 degrees Celsius. After this the sections were dried at 60 degrees Celsius for 45 to 60 minutes. These sections were then deparaffinized in xylene, rehydrated through a graded alcohol series and rinsed with water. For Antigen retrieval the slides were treated in Tris EDTA buffer ph 9.0 at high temperature in microwave for 10× 3 times and the slides were allowed to cool for 10–15 minutes at room temperature. The slides were washed well in distilled water. Endogenous peroxidase was blocked with blocking solution (peroxidase) for five minutes. The slides were rinsed well in distilled water and then with Tris buffer pH 7.6 and then treated with p53 and cytokeratin 20 antibodies for 30 minutes at room temperature. The sections were washed with Tris buffer and treated with Polymer (Envision system K 5007). Colour was developed by DAB. The slides were treated in the DAB solution for 5–7 minutes and then washed well in tap water, counter stained with hematoxylin, dehydrated with graded alcohol, cleared in xylene phenol and xylene. Finally mounted in DPX. Brown nuclear staining was considered positive for p53.

Expression of p53 was calculated as a percentage of labeled nuclei per 500 cells counted in most immunoreactive region of the tumor and categorized into negative, weak positive and strong positive. Negative expression of p53 was reported when <5% of the cells, counted from the most immunoreactive regions of the section show nuclear staining for p53. Positive expression of p53 was considered when >5% of the cells counted from most immunoreactive region of the section show nuclear staining for p53. Positive expression was sub-categorized into weak and strong positive. Weak positive was defined as 5-10% of tumor cells being positive for p53 expression while strong positive is characterized by >10% cells showing positive expression.

Cytokeratin 20 expression was divided into positive and negative expression. Negative expression was defined as cytokeratin 20 staining restricted to superficial cells of the urothelium or less than three cells in intermediate cells of the urothelium. Positive expression was called when immunoexpression was seen in deeper layers of urothelium as clusters of more than three positively stained cells or diffuse staining of urothelium. Positive expression is subcategorized into two categories. Focal positive when less than 10% of the tumor cells were stained in most immunoreactive region of the tumor and diffuse positive when greater than 10% of the tumor cells were stained.

## Results

**Figure 1:**
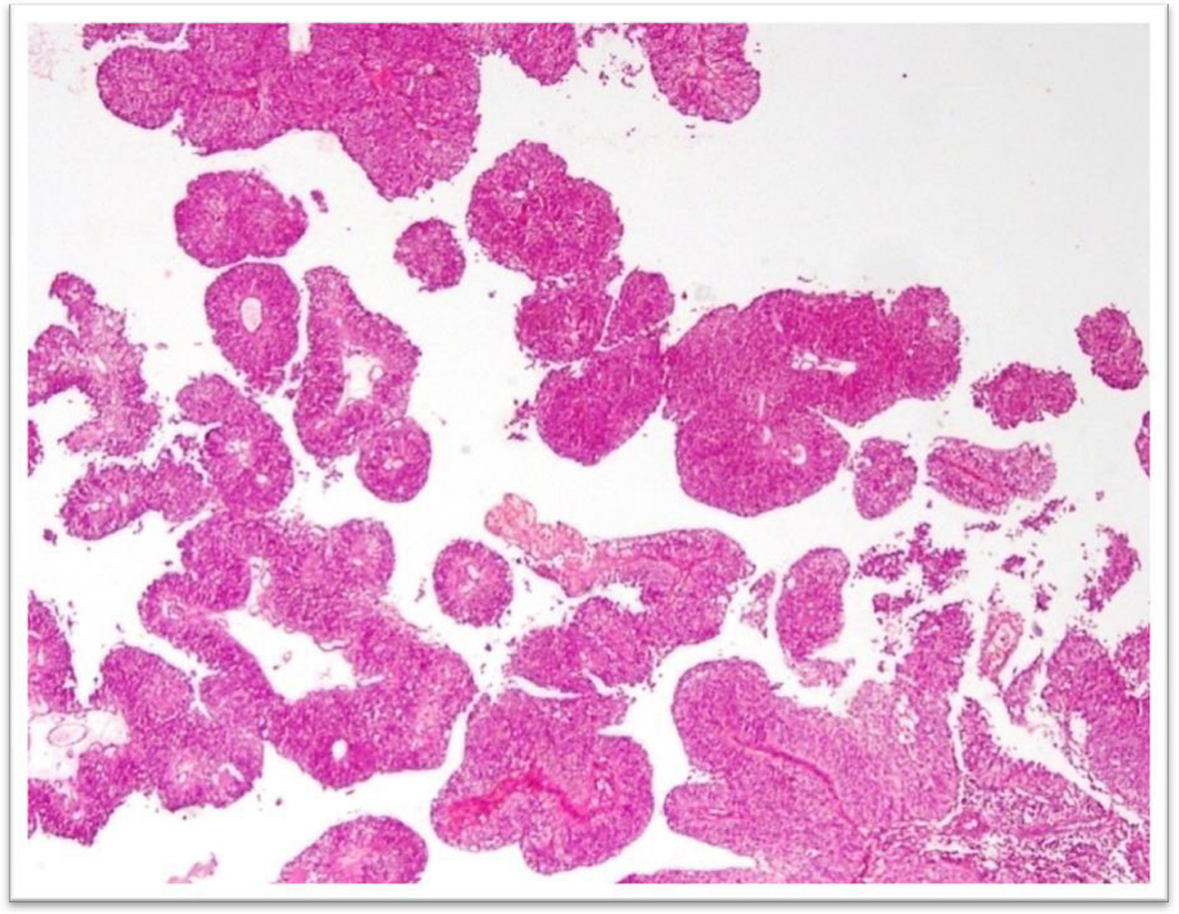
H & E stained sections of low grade Urothelial Carcinoma. Note papillary architecture with focal branching, predominantly ordered and cohesive.

**Figure 2:**
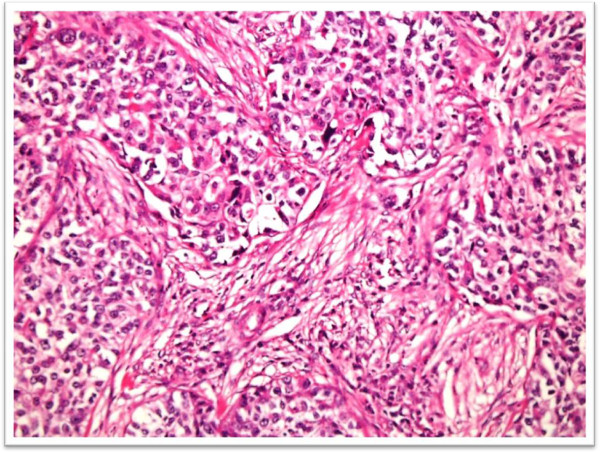
H & E stained sections of high grade Urothelial carcinoma showing muscularis propria invasion.

**Figure 3:**
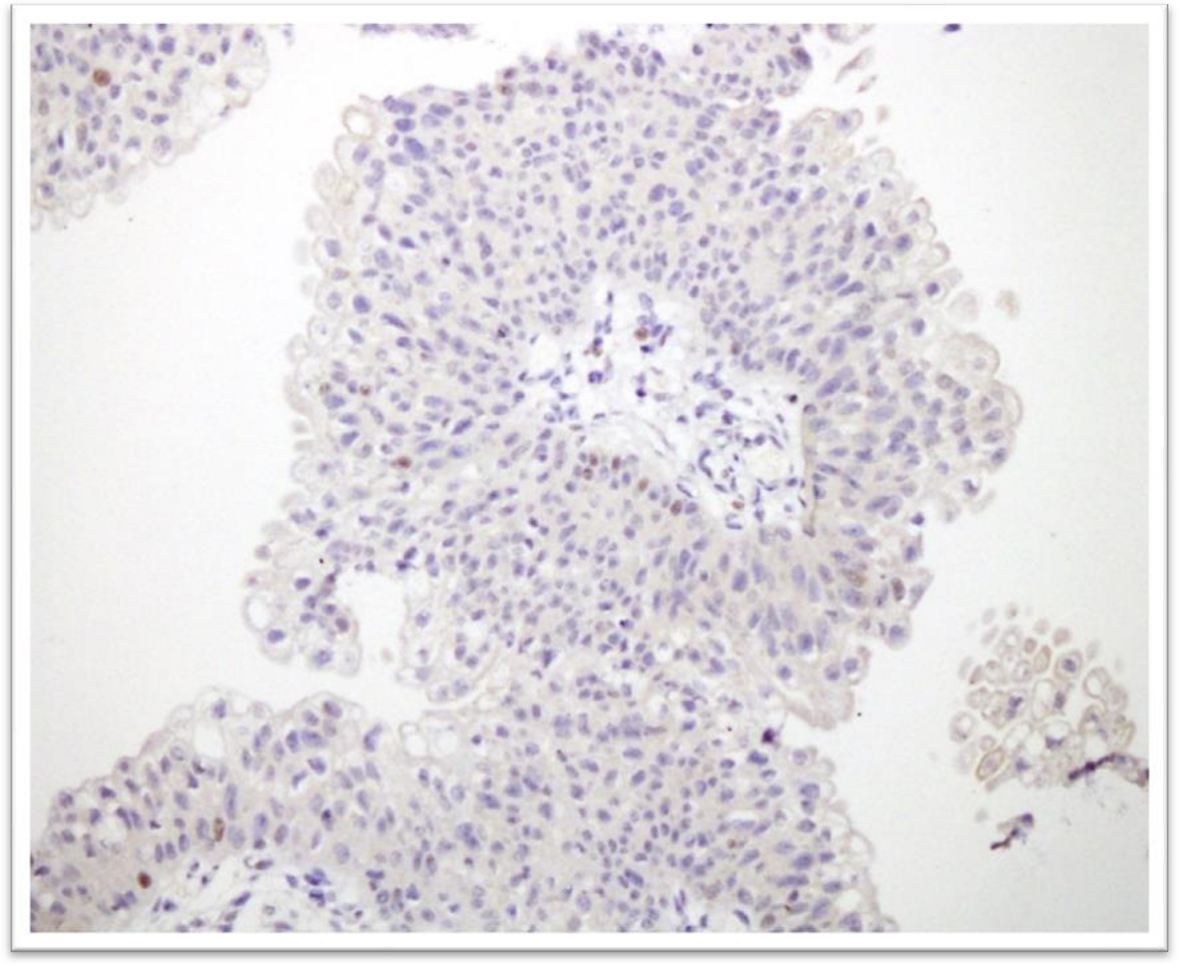
**Cytokerain 20 immunostaining in low grade Urothelial carcinoma.** Positive expression is noted in superficial umbrella cells along with focal positive staining in deeper layers of urothelium.

**Figure 4:**
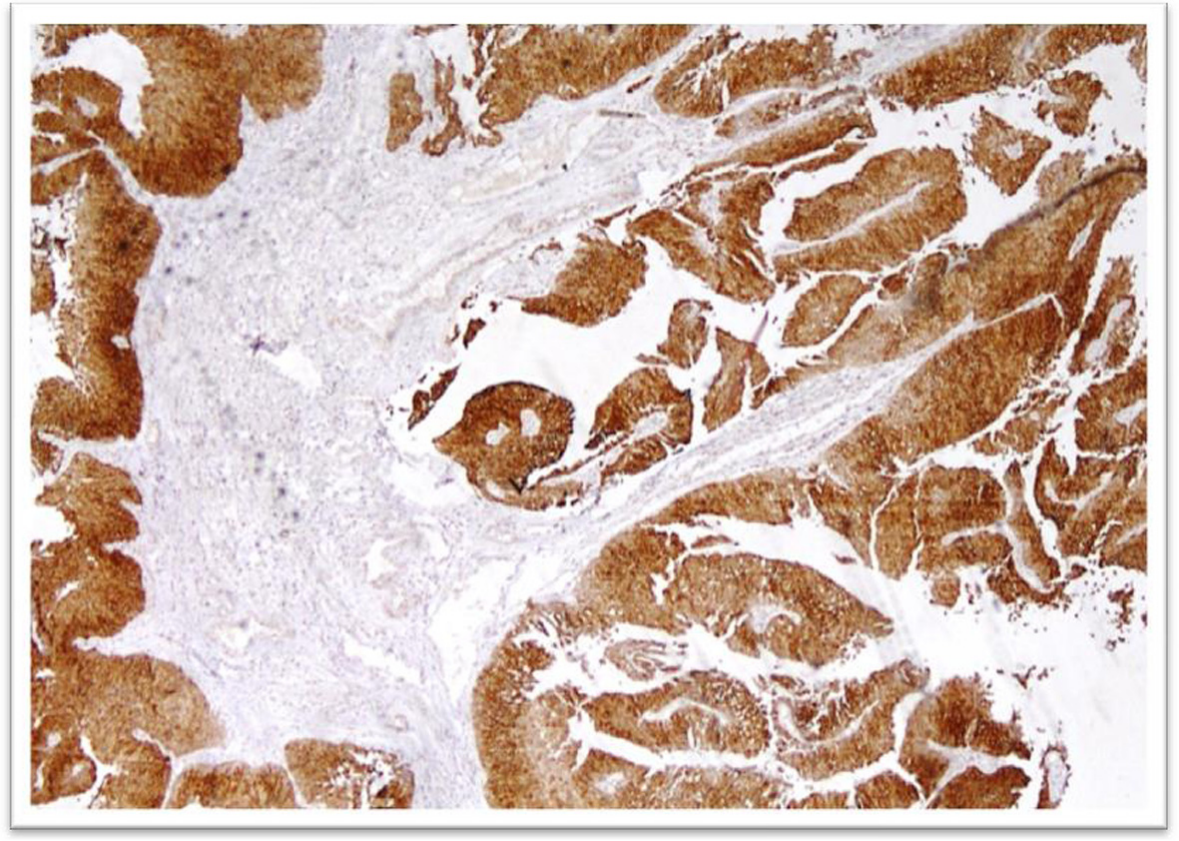
Cytokeratin 20 immunostaining in high grade urothelial carcinoma showing diffuse positive expression.

During the study period, 95 bladder biopsy specimens diagnosed as urothelial carcinomas were identified. There were 48 cases of low grade (Figure 1) and 47 cases of high grade (Figure 2) urothelial carcinomas. 77 cases were seen in males (81.1%) and 18 (18.9%) cases were seen in females with a male to female ratio of 4.2:1. The age of the patients ranged from 22 to 87 years with a mean of 57.8 years. Majority of patients (80%) belonged to 45 to 90 years age group. High grade tumors were predominantly seen at an older age (Table 1). Cytokeratin 20 expression was categorized into negative, focal positive and strong positive (Figures 3 and 4). Significant difference in CK 20 expression was found between low and high grade urothelial carcinomas (Table 2). Diffuse positive expression of cytokerain 20 was seen in 33 high grade tumors (68.8%) and while only 19 low grade tumors (40.6%) showed such positivity.

**Table 1:** Co-relation of Age groups with tumor grade

**Age groups (years)**	**Grade**		**Total**
	**High grade**	**Low grade**	
20-30	0	3	3
31-45	6	10	16
46-90	42	34	76
Total	48	47	95

**Table 2:** Co-relation of Cytokeratin 20 expression with tumor grade

**Tumor grade**		**CK20**			**p-value**
		**Negative**	**Focal positive**	**Diffuse positive**	
High grade	Frequency (%)	4 (8.3%)	11 (22.9%)	33 (68.8%)	
Low grade	Frequency (%)	8 (17%)	20 (42.6%)	19 (40.4%)	0.021^*^
Total	Frequency (%)	12 (12.6%)	31 (32.6%)	52 (54.7%)	

*p-value is significant at <0.05 level.

**Figure 5:**
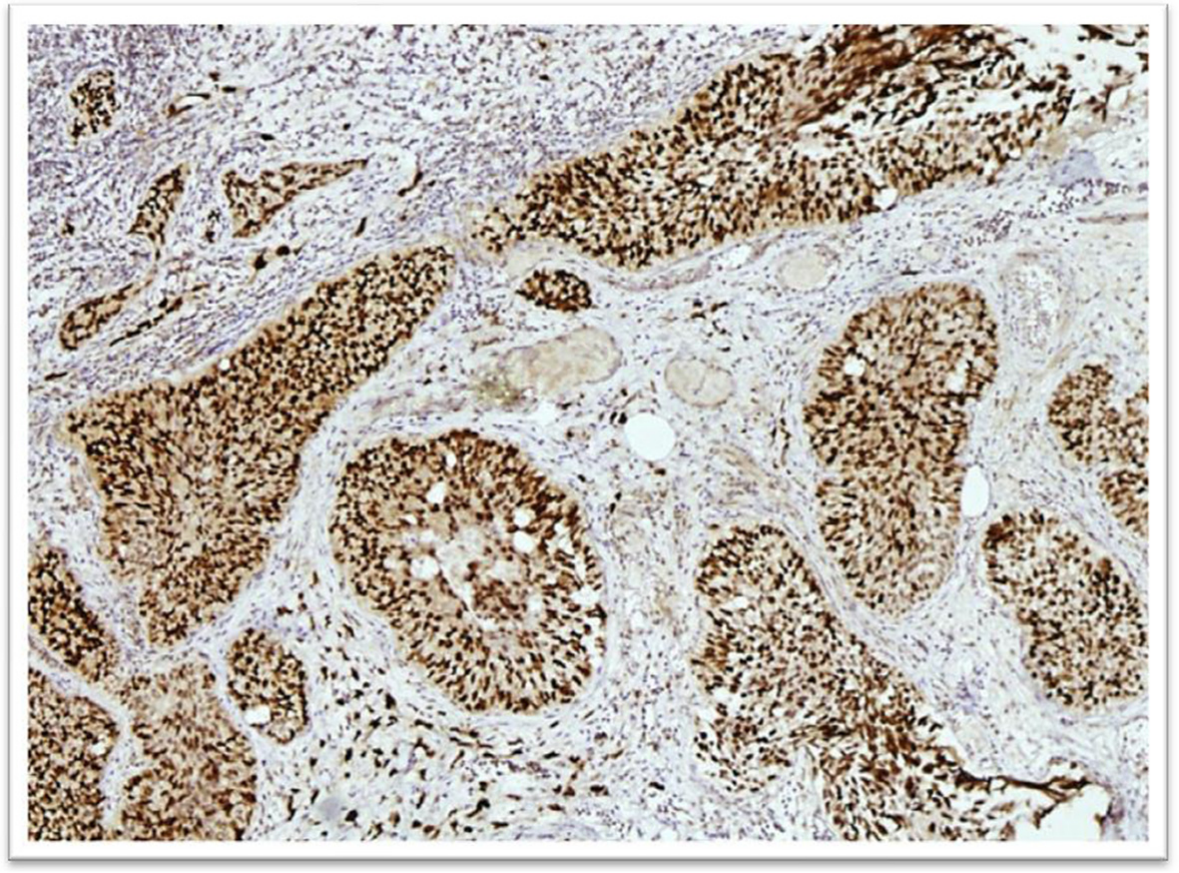
P53 immunostaining in high grade urothelial carcinoma.

**Figure 6:**
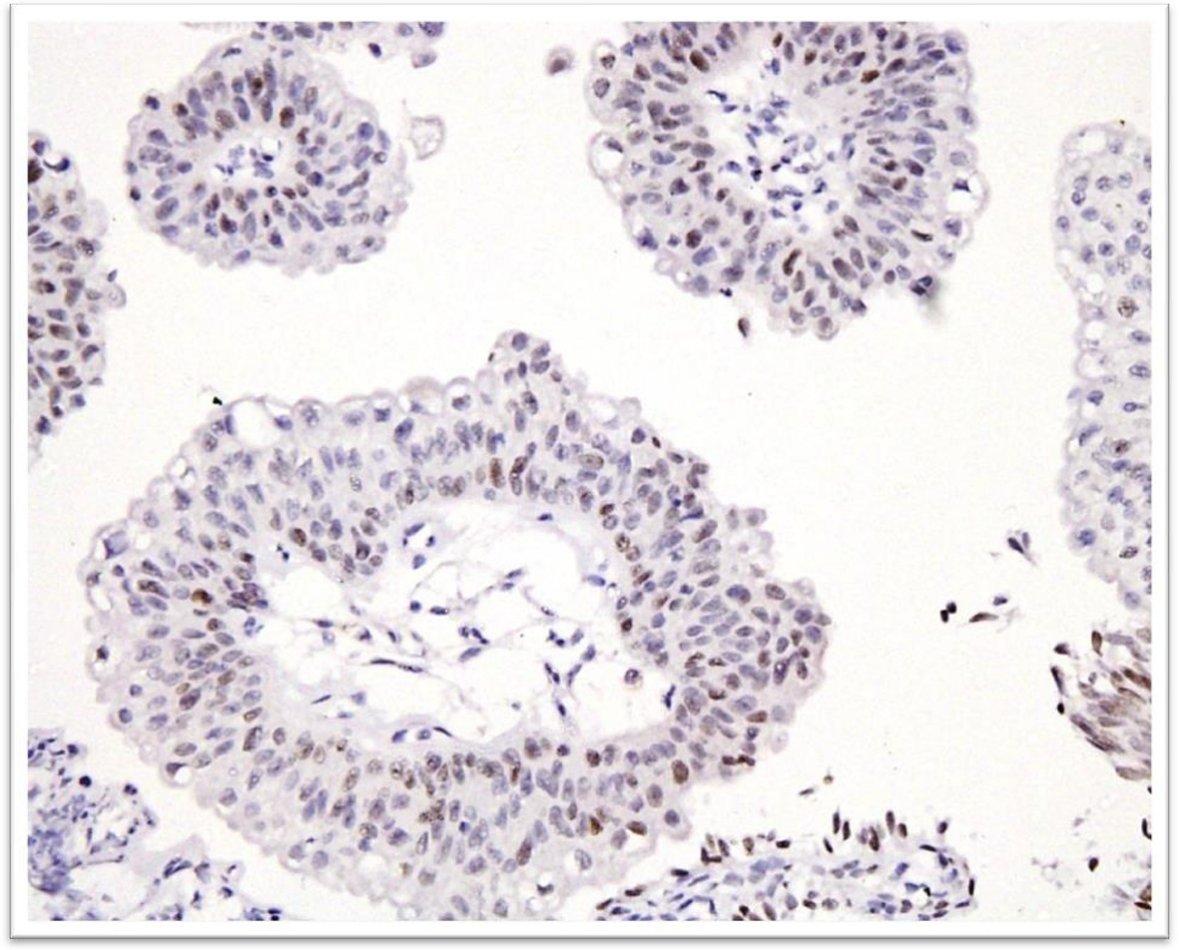
P53 immunostaining in low grade Urothelial carcinoma showing positive expression in 5 to 10% of cells.

Remarkable difference in expression of p53 was noted in two tumor grades (Figures 5 and 6). The strong positive expression of p53 was seen in 35 cases of high grade (72.9%) while only 17 cases (36.2%) of low grade tumors revealed strong expression (Table 3).

**Table 3:** Co-relation of p53 expression with tumor grade

**Tumor grade**		**p53 expression**			**P-value**
		**Negative**	**Weak positive**	**Strong positive**	
High grade	Frequency (%)	(6.3%)	10 (20.8%)	35 (72.9%)	
Low grade	Frequency (%)	5 (10.6%)	25 (53.2%)	17 (36.2%)	0.001^*^
Total	Frequency (%)	8 (8.4%)	35 (36.8%)	52 (54.7%)	

*p-value is significant at <0.05 level.

Out of 95 biopsy specimens, muscularis propria was identified in 70 cases, among which 17 cases revealed muscularis propria invasion. Out of these 17 cases of invasive urothelial carcinoma, 15 and 11 cases showed P53 and CK20 expression respectively. However, a high percentage of non-invasive carcinomas also expressed these markers as shown in Tables 4 and 5.

**Table 4:** Co-relation of p53 expression with muscularis propria invasion

**P53 expression**	**Muscularis propria invasion**			**P-value**
	**Present**	**Absent**	**Muscularis propria not present**	
Negative	2 (2.8%)	5 (7.1%)	1	
Weak positive	0 (0%)	22 (31.4%)	13	0.006^*^
Strong positive	15 (21.4%)	26 (37.1%)	9	
Total	17 (24.3%)	53 (75.7%)	23	

*p-value is significant at <0.05 level.

**Table 5:** Co-relation of CK20 expression with muscularis propria invasion

**CK20 expression**	**Muscularis propria invasion**			**P-value**
	**Present**	**Absent**	**Muscularis propria not present**	
Negative	2 (2.8%)	9 (12.8%)	1	
Focal positive	4 (5.7%)	15 (21.4%)	12	0.18^*^
Diffuse positive	11 (15.7%)	29 (41.4%)	10	
Total	17 (24.3%)	53 (75.7%)	23	

*p-value is not significant at <0.05 level.

Combined expression of P53 and CK20 was seen in 40% of high grade and 22% of low grade carcinomas (Table 6) while 14 cases of invasive carcinoma showed combined P53 and CK20 expression (Table 7).

**Table 6:** Co-relation of combined expression of P53 and CK20 with tumor grade

**Combined P53 and CK20 expression**	**Grade**		**P-value**
	**High grade**	**Low grade**	
Positive	37 (40%)	21 (22%)	
Negative	11 (11.5%)	26 (27.3%)	0.001^*^
Total	48 (50.5%)	47 (49.5%)	

*p-value is significant at <0.05 level.

**Table 7:** Co-relation of combined expression of P53 and CK20 with muscularis propria invasion

**Combined P53 and CK20 expression**	**Muscularis propria invasion**			**P-value**
	**Present**	**Absent**	**Muscularis propria not present**	
Positive	14 (20%)	33 (47%)	9	
Negative	3 (4.3%)	20 (28.6%)	14	0.02^*^
Total	17 (24.3%)	53 (75.7%)	23	

*p-value is significant at <0.05 level.

Higher expression of P53 and CK 20 (38% combined expression) was found in males as compared to females (14.3% combined expression). Similarly highest expression of P53 and CK20 was seen in older age group of above 45 years (44% combined expression). This reflects aggressive tumor behavior in older men.

## Discussion

P53, is a nuclear phosphoprotein which acts as tumor suppressor and plays a role in apoptosis, genetic stability, and inhibition of angiogensis. Wild-type p53 protein has a short half-life; however, the protein encoded by mutated p53 remains active for a long period. Therefore, mutation of p53 gene results in p53 accumulation in cells nuclei. This accumulation is detectable with immunohistochemical methods and correlates with p53 gene mutation, thus, detection of p53 protein in the nuclei of cells by immunohistochemical methods.

The cytokeratins are the intermediate filament proteins characteristic of epithelial cells. Some 20 different cytokeratin isotypes have been identified. Epithelial cells express between two and ten cytokeratin isotypes and the consequent profile which reflects both epithelial type and differentiation status may be useful in tumour diagnosis. The transitional epithelium or urothelium of the urinary tract shows alterations in the expression and configuration of cytokeratin isotypes related to stratification and differentiation. The most important recent finding is the demonstration that a normal CK20 expression pattern is predictive of tumor non-recurrence and can be used to make an objective differential diagnosis between transitional cell papilloma and carcinoma. Its expression beyond superficial layers of urothelium is considered positive [11,13,14].

There are various international studies which have compared p53 expression in various grades of urothelial tumors along with different parameters and in combination with various new immunohistochemical markers. In a study conducted by Yin H, who applied p53, cytokeratin 20 and Ki-67 on 84 noninvasive papillary urothelial tumors graded by the 1973 WHO and 1998 WHO/ISUP classifications and compared the expression of these markers along with morphological parameters using both classification systems to validate the recent grading system in application [15]. Using recent WHO/ISUP classification two third of low grade carcinoma (34 out of 53 cases) i.e. 64% and all 19 cases of high grade carcinoma showed a diffuse staining pattern for cytokeratin 20. The remaining low grade carcinomas showed focal staining pattern. On the other hand p53 was not expressed in low malignant potential cases. The p53 index of greater than 10% was observed in only 5 cases of low grade carcinomas, and in 42 of the 53 cases i.e. 79% the p53 expression was less than 5% and was considered negative.

In another study conducted by Toktas in Turkey, correlated nuclear p53 accumulation with prognosis, they include total 90 patients of urothelial carcinomas, using old version of WHO classification and found that those tumors which expressed p53 had higher rate of recurrence and progression and shorter survival [16].

In another study by Tsai et al. the clinical significance of p53 and Her-2/neu expression was evaluated [16]. They included 67 patients with invasive bladder cancer who have gone through radical surgery. The positive staining for p53 and Her-2/neu expression was seen in 44.8% and 39% patients. The expression of both the markers was significantly associated with higher tumor grades and pathologic stage along with the lymph nodes status.The patients showing greater co expression of both the markers had the worse prognosis. Similarly, Yildiz et al. used dual cocktail immunostain for p53 and cytokeratin 20 for diagnosis of non neoplastic and neoplastic bladder biopsies [17]. The dual positivity for both of these markers was mainly seen in carcinoma in situ and carcinomas while cases showing reactive atypia or mild dysplastic changes were negative or showed focal staining mainly in the superficial urothelium.

Our study also validates the results of Sangeeta Desai et al., who performed cytokeratin 20 on 42 low grade and 62 high grade urothelial carcinomas [17]. They found cytokeratin 20 positivity was associated with increasing tumor grade and stage. They found that 69.4% cases of high grade tumors were showing cytokerain20 positivity compared to 45% of tumors of low grade category.

We also evaluated the co-relation of P53 and CK20 expression with muscularis propria invasion which is one of the prime prognostic factors in urothelial malignancies. Although 14 out of 17 cases which were muscle invasive exhibited combined expression of these markers, however these markers cannot be used as surrogate markers for muscle invasion as 47% of non-invasive urothelial carcinomas also showed co-expression of these markers.

As histologic grading parameters are not absolute, therefore several investigators evaluated the role of adjunctive markers apart from p53 and CK20. In a recent study involving 193 cases of non-invasive urothelial malignancies, assessed the value of proliferation markers in assigning grade to non-invasive urothelial carcinoma. They found the positive predictive value of Ki67 and phosphohistone H3 to be 0.15, which is comparable to that of WHO [18].

In another study immunohistochemical expression of Her2neu and EGFR were found to be positively associated with poor differentiation and advanced stage [19].

Chen YB et al. in a study involving 51 bladder biopsies found significant inter-observer variability in grading urothelial carcinoma among 5 pathologists and therefore they recommended use of survivin as a useful adjunctive tool. Survivin score outperformed Ki-67 in separating the high-grade group from the low-grade group and showed a significantly higher predictive accuracy for high-grade recurrence [20].

We did not performed these markers on cases with the diagnosis of papilloma and papillary urothelial neoplasms of low malignant potential as morphologically they lack fused and branching papillary architecture seen in low and high grade urothelial carcinoma. Moreover they don’t show cytologic atypia and mitotic activity, therefore there categorization is not difficult. On the other hand segregation of low and high grade carcinomas can be very difficult at times specially in limited tissue specimens. More-over risk factor assessment like exposure to carcinogens was not done as detailed history was not available.

We found that evaluation of p53 expression is relatively easy to interpret in urothelial malignancies, as p53 is a nuclear stain while sometimes interpretation of CK 20 expression may be difficult. More over more combined expression of P53 and Ck20 is especially useful in assigning grade to urothelial malignancies in difficult situations as histologic reproducibility of tumor grade is poor. Therefore we suggest that p53 with or without CK20 should be used as an adjuctive tool in grading urothelial malignancies when the histologic features are borderline.

## Conclusion

The p53 and cytokerain 20 expression is diffuse and strong positive in cases of higher grade urothelial carcinoma as compared to low grade urothelial carcinoma which in majority of cases show focal and weak positive expression. These markers should be used in assigning grade to urothelial malignancies in cases of borderline histologic features.

## Competing interests

The authors declare that they have no competing interests.

## Authors’ contributions

SM: main author of manuscript, have made substantial contributions to conception and design of data.AAA: main author of manuscript, have made substantial contributions to analysis and interpretation of data. SHH: have given final approval of the version to be published. MME: have been involved in drafting and revision of the manuscript. MK: have been involved in drafting the manuscript. All authors read and approved the final manuscript.
